# Enhancement of Titania Photoanode Performance by Sandwiching Copper between Two Titania Layers

**DOI:** 10.3390/ma13194326

**Published:** 2020-09-28

**Authors:** Fan Yang, Ruizhuang Yang, Lin Yan, Jiankun Wu, Xiaolin Liu, Lirong Yang, Minglong Zhong, Xuan Luo, Lin Zhang

**Affiliations:** 1 Research Center of Laser Fusion, China Academy of Engineering Physics, Mianyang 621900, China; yangf711@mail.ustc.edu.cn (F.Y.); rzyang99@mail.ustc.edu.cn (R.Y.); yanlin77@mail.ustc.edu.cn (L.Y.); wujiankun19@163.com (J.W.); lxl91003@mail.ustc.edu.cn (X.L.); yanglirong83@163.com (L.Y.); 13110200008@fudan.edu.cn (M.Z.); zhanglin@caep.cn (L.Z.); 2 Department of Physics, University of Science and Technology of China, Hefei 230026, China

**Keywords:** metal reduction, oxygen vacancies, TiO_2_, electronic structure, photoanode

## Abstract

Vacancies in semiconductors can play a versatile role in boosting their photocatalytic activity. In this work, a novel TiO_2_/Cu/TiO_2_ sandwich structure is designed and constructed. Abundant vacancies were introduced in TiO_2_ lattice by Cu reduction under heat treatment. Meanwhile, Cu atom could diffuse into TiO_2_ to form Cu-doped TiO_2_. The synergistic effect between oxygen vacancies and Cu atoms achieved about 2.4 times improved photocurrent of TiO_2_/Cu/TiO_2_ sandwich structure compared to bare TiO_2_ thin film. The enhanced photoactivity may be attributed to regulated electron structure of TiO_2_ by oxygen vacancies and Cu dopant from experimental results and density functional theory calculations. Oxygen vacancies and Cu dopant in TiO_2_ formed through copper metal reduction can introduce impurity levels and narrow the band gap of TiO_2_, thus improve the visible light response. More importantly, the Cu^2+^ and oxygen vacancies in TiO_2_ lattice can dramatically increase the charge density around conduction band and promote separation of photo-induced charge carriers. Furthermore, the oxygen vacancies on the surface may serve as active site for sufficient chemical reaction. This work presents a novel method to prepare doped metal oxides catalysts with abundant vacancies for improving photocatalytic activity.

## 1. Introduction

Photoelectrochemical (PEC) water splitting is a promising strategy to convert renewable solar energy into chemical energy for tackling the growing energy shortage and environmental crisis. In comparison with other energy conversion approaches, PEC water splitting stands out due to its low cost, environmental friendliness and easy separation of hydrogen and oxygen from water [1,2]. Unsatisfactorily, the energy conversion efficiency is hindered by the sluggish reaction kinetics which involves four electrons oxidation process [3,4]. Many studies have focused on the photoanode for accelerating the water oxidation reaction [5,6]. Many semiconductors have been investigated as photoanodes. Among them, TiO_2_ is still a promising candidate because of its appropriate band edge position, abundant reserves and excellent chemical stability. However, the water oxidation kinetics of TiO_2_-based photoelectrodes is severely impeded by wide band gap and fast electron−hole recombination. To solve these problems, great efforts have been made on TiO_2_ materials. A common and effective method is introducing extra elements into TiO_2_ crystal. Metal ions such as Cu [7], Mg [8], Sn [9] and Fe [10,11] have been doped to regulate the electron structure of TiO_2_. Typically, a transition metal would create donor levels near the conduction band minimum by their 3d orbital electrons. Many studies have experimentally proved that the doped metal elements would red shift absorption edge of TiO_2_ to visible light region, which would greatly improve the photocatalytic performance. Zhang and co-authors [12] tuned the absorption edge of TiO_2_ up to 700 nm by incorporating Cu element to TiO_2_ nanosheet. The resulted Cu-doped TiO_2_ nanosheet exhibited remarkable photoactivity. In addition, non-metal elements have been explored to dope in TiO_2_ materials [13,14,15]. The substitutional non-metal elements would create the acceptor state above the valence band maximum, leading to band gap narrowing and an expansion of photo-absorption region. However, the non-metal ions could also serve as color center to facilitate the recombination of charge carriers to some extent.

As a dopant-free strategy, oxygen vacancies (V_o_) play a crucial role in metal oxide semiconductors. [16,17,18,19,20,21] The previous studies indicate that oxygen vacancies could introduce defect level in semiconductor’s band gap and promote the exciton dissociation as well. The defects on the surface of semiconductors may act as active sites for chemical reaction. Thus, V_o_-rich semiconductor exhibits enhanced photocatalytic activity due to its improved light absorption, charge separation ability and surface reaction. Chen and co-authors [22] firstly reported the V_o_-rich TiO_2_ prepared by H_2_ reduction, and revealed the important impact of oxygen vacancies on the structure and properties of TiO_2_ material. Since that, a series of strategies such as metal (e.g., Al) reduction [23], UV radiation [24] and plasma treatment [25,26] are developed to create V_o_ in metal oxides materials. The melted Al is used as a reductant to decrease oxygen partial pressure and drive TiO_2_ reduction kinetically. At the same time, the melted Al will also be partially oxidized with formation of enough oxygen vacancies. It may be a promising and effective strategy to prepare V_o_-rich heterostructure and V_o_-rich materials with doped metal through metal reduction approach. There is rare study combining metal doping with metal reduction. One challenge is metal nanoparticles, especially the 3d transitional metal, are easily oxidized to metal oxides when exposed to air or oxygen atmosphere because the nanoparticles always possess strong redox ability. Coating with chemically stable material could prevent the active metal from oxidation, and the stability is thereby increased [27]. Previous study [28] reported the Cu nanoparticles embedded in TiO_2_ particles for CO_2_ reduction. However, the fabrication process was complicated and strict.

We designed and prepared TiO_2_/Cu/TiO_2_ films with sandwich structure. After annealing under Ar atmosphere, the core cooper layer was reduced surrounding TiO_2_ films and cooper atoms also diffused into TiO_2_ lattice. The resulted material possessed abundant oxygen vacancies with copper dopants.

## 2. Materials and Methods

### 2.1. Deposition of TiO_2_/Cu/TiO_2_ Thin Films

R200 ALD reactor (Picosun, Espoo, Finland) with thermal reaction chamber was employed to deposit TiO_2_ on fluorine-doped tin oxide (FTO, Foshan shi yuan jing mei glass Co. Ltd., Foshan, China) substrate [29] The titanium tetrachloride (TiCl_4_, 99.999%, Nanjing ai mou yuan Scientific equipment Co., Ltd., Nanjing, China) and ultra-pure water (H_2_O) were selected as Ti and O precursors, respectively. The ultrahigh purity N_2_ served as carrier gas and purge gas during the deposition process. The 600 cycles of TiO_2_ deposition were performed at 160 °C. Typically, the pulse times for TiCl_4_ and H_2_O were 1 s and 0.1 s, respectively.

Cu thin film was deposited on as-prepared TiO_2_/FTO by magnetron sputtering system [30] (SKY technology development, Shenyang, China). In brief, as-prepared TiO_2_/FTO samples were immediately transferred into the deposition chamber. A copper target (99.95% purity) was employed in the deposition chamber with a base pressure of 5.0 × 10^−3^ mbar. The sputtering power was kept at 40 W for all samples. The deposition time was 1, 2, 3 and 4 min to obtain copper films with different thicknesses, respectively. After that, Cu/TiO_2_/FTO substrates were taken out and quickly delivered to ALD reaction chamber. Next, in 600 successive cycles, TiO_2_ was deposited on Cu/TiO_2_ FTO substrates for the formation of TiO_2_/Cu/TiO_2_ (denoted as TCT) sandwich structure. After being calcined at 500 °C for 2 h under Ar atmosphere, the samples were denoted as TCT-1, TCT-2, TCT-3 and TCT-4, respectively.

### 2.2. Density Functional Theory Calculation

The density of states and electronic band structure were estimated by CASTEP module in Materials Studio. The 2 × 2 × 1 supercells of TiO_2_ with no vacancy in lattice (perfect TiO_2_), TiO_2_ with oxygen vacancies, TiO_2_ with Cu^2+^ and TiO_2_ with incorporation of Cu^2+^ and Cu^+^ structures were built to perform the DFT + U calculations. For Ti 3d orbitals, the Hubbard U correction was set to 5.9 eV [31]. GGA (Generalized gradient approximation)-PBE (Perdew–Burke–Emzerhof) exchanged function and ultrasoft pseudopotential was used to correct the exchange correlations between electrons. Ionic relaxations were optimized under conventional energy with 2 × 10^−5^ eV and all atoms were allowed to relax until forces were less than 0.05 eV/Å. The plane wave cutoff energy was set to 450 eV.

### 2.3. Characterization

The thicknesses of ALD TiO_2_, Cu layers and TiO_2_/Cu/TiO_2_ thin films were determined by ellipsometry measurement (SENTECH SE850, Sentech, Berlin, Germany). Measurements were conducted at 70° angle of incidence with wavelengths ranging from 400 to 800 nm. Field-emission scanning electron microscope (FE-SEM, ZEISS ultra 55, Lel Tech, Jena, Germany) with an accelerating voltage of 20 kV was utilized to observe the morphologies of these TiO_2_/Cu/TiO_2_ materials. The phase structure and crystallinity were revealed by X-ray diffraction (XRD, D8 advance system, Bruker, Billerica, MA, USA) with a copper Kα radiation (λ = 1.5406 Å). Transmission electron microscopy (JEOL-2100F, Jeol, Akishima, Japan) with energy dispersive X-ray spectroscopy (Quantax 1.9, Bruker Nano GmbH, Berlin, Germany) with an acceleration voltage of 200 kV was used to observe the morphology and element dispersion. X-ray photoelectron spectroscopy (XPS) and Auger spectra measurement was performed on ESCALAB 250Xi (ThermoFischer, Waltham, MA, USA) system with Mg Kα radiation at a pressure of about 5 × 10^−10^ mbar. The optical absorption was investigated by UV–vis absorbance spectra (LS 5 Lambda 950, PekinElemer, Waltham, MA, USA) from 200 to 800 nm. InVia Reflex (Renishaw, *Gloucestershire*, UK) microscope was employed to record Raman spectra under excitation wavelength at 532 nm. Photoluminescence (PL) spectra were recorded on a PekinElemer LS55 spectrofluorometer at room temperature under different excitation wavelength at 390 and 425 nm, respectively.

### 2.4. Photoelectrochemical Measurement

The PEC measurements were performed on a CHI 660E electrochemical station (Shanghai Chenghua Machinery Company, Shanghai, China). A standard three-electrode quartz cell with 0.5 M Na_2_SO_4_ aqueous solution (pH = 6.8) was employed. The as-prepared TCT samples on FTO substrate were treated as work electrodes. Platinum foil and saturated calomel electrode were used as counter and reference electrode, respectively. A 300-W Xe lamp (PLS-SXE300/300UV, PerfectLight, Beijing, China) with optical filter was used as solar-simulated light source. The photocurrent responses of the photoelectrodes were conducted by linear sweep voltammetry (LSV) at a scan rate of 10 mV s^−1^ under chopped light illumination. The electrochemical impedance spectroscopy (EIS) was recorded by applying AC voltage amplitude of 5 mV with frequency ranging from 0.1 Hz to 100 kHz under light irradiation. Incident photon to current efficiency (IPCE) tests were performed by LSV under Xe lamp irradiation coupled with a monochromator from 300 to 550 nm. Mott–Schottky plots were obtained by applying DC potential range at a frequency of 1 kHz.

## 3. Results and Discussion

### 3.1. Characterizations of TiO_2_/Cu/TiO_2_ Films

#### 3.1.1. SEM Micrographs

**Figure 1:**
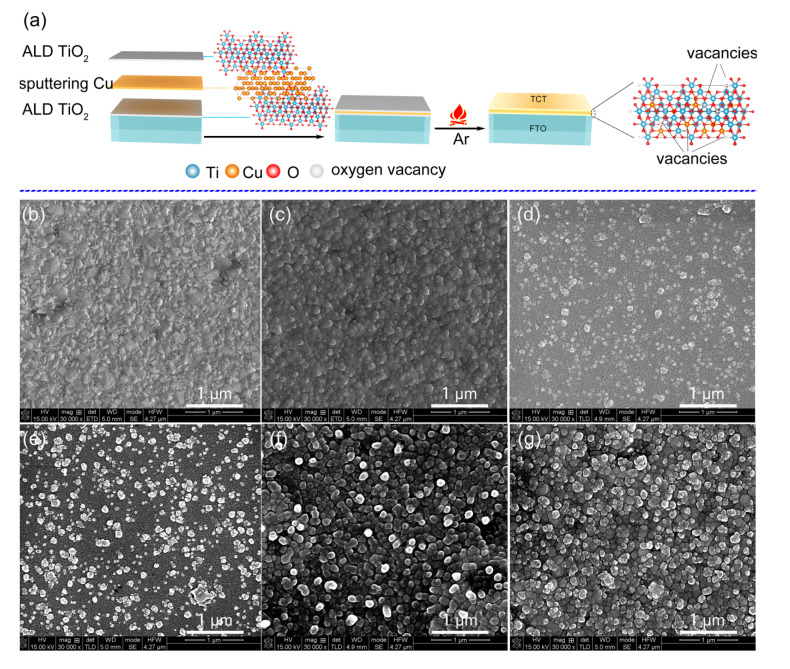
Schematic illustration of preparation process of Cu-doped TiO_2_ thin films by Cu reduction (**a**); and SEM images: of FTO glass (**b**); bare TiO_2_ (**c**); TCT-1 (**d**); TCT-2 (**e**); TCT-3 (**f**); and TCT-4 (**g**).

The preparation of Cu-doped TiO_2_ films with abundant oxygen vacancies is illustrated in Figure 1a. Cu embedded in TiO_2_ double layers with sandwich structure was achieved through ALD and magnetron sputtering technique. After heat treatment under Ar atmosphere, the Cu layer could reduce surrounding TiO_2_ films and Cu atoms diffused into TiO_2_ lattice. As shown in Table 1, the thicknesses of as-prepared bare TiO_2_ and TCT thin films increased from 104.74 to 144.59 nm with the sputtering time prolonging. The morphology of TiO_2_ thin film with/without Cu layer embedded was characterized by SEM measurement. As displayed in Figure 1, the bare TiO_2_ thin film had a similar appearance with FTO substrate, suggesting the conformal deposition of TiO_2_ on the substrate by atomic layer deposition [29]. After sputtering the cooper films, the extra nanoparticles appeared on the surface, which indicated island growth of cooper film by magnetron sputtering. Besides, the longer sputtering time resulted in denser nanoparticles.

#### 3.1.2. X-Ray Diffraction

**Figure 2:**
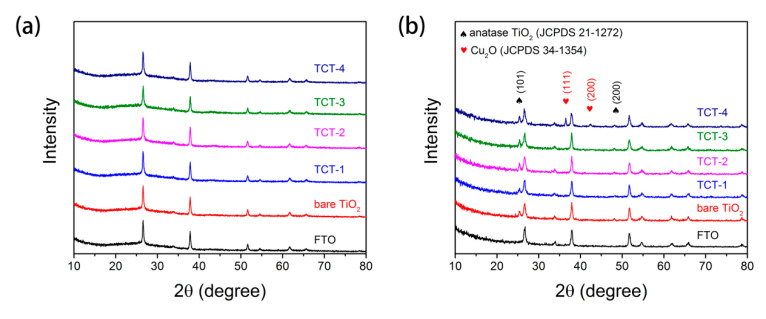
XRD pattern of FTO, bare TiO_2_ and different TCT samples before (**a**) and after (**b**) annealing.

**Figure 3:**
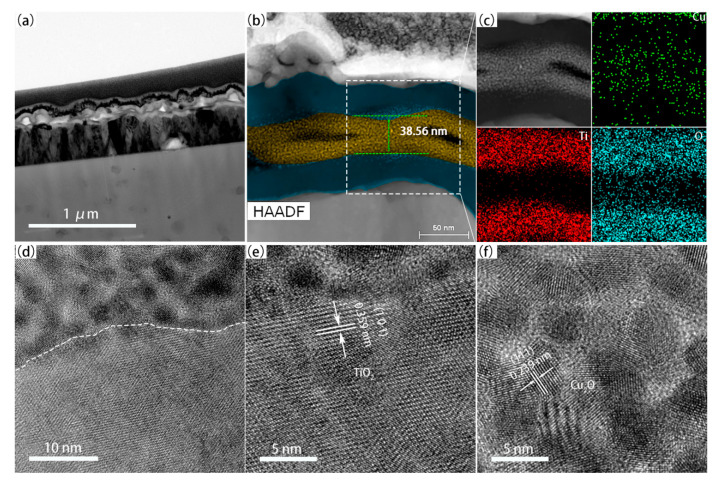
Cross-sectional TEM image with low magnification ((**a**) 1 μm and (**b**) 50 nm); EDX image (**c**); and high resolution TEM images at the interface (**d**) between TiO_2_ layer (**e**) and Cu layer (**f**) of TCT-4.

The XRD pattern in Figure 2a suggests that the as-prepared TCT materials without heat treatment showed no identified phase of copper or copper oxides. It indicated that the deposited metallic Cu layers had poor crystallinity and good stability in air [32] As displayed in Figure 3b, all TiO_2_ samples with Cu embedded had typical anatase TiO_2_ phase (JCPDS No. 21-1271) after annealing process. Notably, extra diffraction peaks appeared in TCT-4 at 38.4 ° and 43.1 °, which were identified as (1 1 1) and (2 0 0) planes of Cu_2_O (JCPDS No. 05-0667). This represented that the core Cu layer was partially oxidized in TiO_2_ shell. There was no obvious Cu_2_O, CuO or Cu phase in other TCT materials, meaning the Cu films were oxidized and diffused into TiO_2_ layers with no or less residual Cu. Previous studies [30,33] reported Cu layer in TiO_2_/Cu/TiO_2_ sandwich structure displaying metallic Cu diffraction peak under 350 °C treatment. It was assumed that metallic Cu could not react with surrounding TiO_2_ layer at such low temperature. The higher concentration of Cu would lead to insufficient reaction of Cu film with TiO_2_ surrounding layers under heat treatment, resulting in the formation of cuprous oxide.

#### 3.1.3. TEM Micrographs

To demonstrate the interaction between Cu and TiO_2_ layers, TEM images of TCT-4 were characterized. The sandwich structure of TCT-4 can be clearly seen in the low-resolution images in Figure 3a,b. The thickness of Cu layer was estimated to be 38.56 nm, which corresponded to the ellipsometry measurement result (39.85 nm). This indicated that the deposition rate was about 10 nm/min during magnetron sputtering process. In addition, the growth rate of ALD TiO_2_ was determined to be 0.92 nm/cycle from the ellipsometry measurement and line scanning model of TEM (Figure S1). As displayed in Figure 3c, Cu atoms diffused into TiO_2_ layer after annealing, and the resident Cu atoms were oxidized with the appearance of oxygen atoms. Obviously, there is a boundary between the TiO_2_ and Cu layer in Figure 3d. The high revolution TEM images in Figure 3e,f show that the lattice spacing is 0.359 nm, ascribed to (1 0 1) plane of anatase TiO_2_. The compound formed in Cu layer was identified as Cu_2_O with the lattice spacing 0.24 nm of (1 1 1) plane, which was consistent with XRD results.

#### 3.1.4. X-Ray Photoelectrons Spectra and Auger Spectra

**Figure 4:**
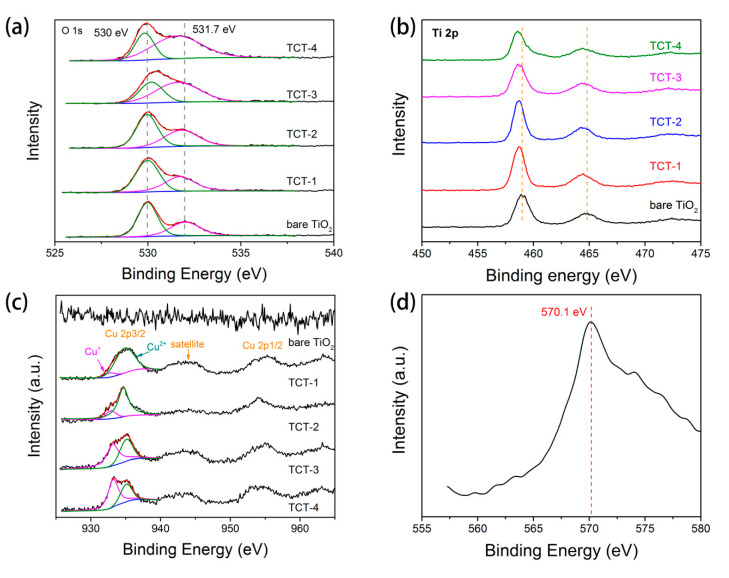
(**a**) O 1s; (**b**) Ti 2p; and (**c**) Cu 2p XPS spectra of bare TiO_2_ and TCT samples; and (**d**) auger spectra of TCT-4 photocatalyst.

To further explore the Cu species and element information of this sandwich structure, the XPS and Auger spectra were employed. Figure 4 and Figure S2 show the O 1s, Ti 2p and Cu 2p core level spectra of bare TiO_2_ and four TCT samples, respectively. It was clearly seen that the peak for O 1s (Figure 4a) could be deconvoluted into two peaks at around 530 and 531.7 eV. These peaks were assigned to the Ti-O bond and oxygen atoms in the vicinity of vacancy [21,29]. With the thickness of Cu film increasing, the concentration of oxygen vacancies grew. This revealed that more metallic cooper reacted with surrounding TiO_2_ films and accelerated the formation of oxygen vacancies. The Ti 2p spectra are displayed in Figure 4b. Compared to bare TiO_2_, the peak position of TCT films showed a negative shift. This shift could be ascribed to formation of Ti^3+^, as the evidence of TiO_2_ reduction [34,35]. Furthermore, The Cu species in TCT materials were revealed by Cu 2p XPS spectrum and auger electron spectra. As exhibited in Figure 4c, all TCT samples had strong Cu signals. The binding energy of Cu 2p3/2 at 935.2 eV could be ascribed to Cu^2+^ species. The satellite peaks at about 943 and 962 eV were evidence of existence of Cu^2+^. The strong satellite peak of TCT-1, TCT-2 and TCT-3 suggested the Cu^2+^ was the main valence state. The binding energy of Cu 2p1/2 at 952.2 eV and Cu 2p3/2 at 932.9 eV could be ascribed to Cu^0^ or Cu^+^. It was difficult to identify Cu^+^ and Cu^0^ due to the small difference between their binding energy. Considering that, the Cu Auger electron spectrum was collected to further confirm the Cu chemical state. The binding energy of Cu LMM is about 570.1 eV, representing the existence of Cu^+^ rather than Cu^0^ (567.9 eV) [36]. This meant that metallic Cu totally reacted with TiO_2_ under annealing process. In addition, the thickness of metallic Cu film determined the interaction between Cu core layer and TiO_2_ shell. Thicker Cu film in TCT-4 would be preferably oxidized to Cu^+^, and thinner film would result in more formation of Cu^2+^, which was in agreement with the XRD results.

#### 3.1.5. Raman Spectroscopy

**Figure 5:**
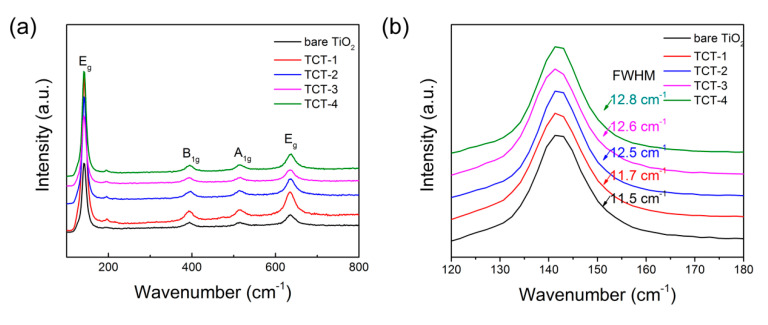
(**a**) Raman spectra of bare TiO_2_ and TCT materials; and (**b**) the enlarged Raman spectra at around 144 cm^−1^ of all five samples.

Raman spectra of different TCT samples and bare TiO_2_ are shown in Figure 5. It was clear that anatase was the main crystalline phase in all samples. There were no obvious signals of Cu or cooper oxides, indicating the small concentration or small grain size of cooper compounds. The vibration Raman modes occurring at 144, 396, 515 and 638 cm^−1^ were attributed to E_g_, B_1g_, A_1g_ and E_g_, respectively [37]. The evidence of Cu doped into TiO_2_ could be identified by analyzing the main vibration mode of anatase at 144 cm^−1^. This E_g_ vibrational mode showed an increasing full width at half maximum (FWHM) from 11.5 to 12.8 cm^−1^ with the Cu content raising. The increasing FWHM can be ascribed to structure regulation related to shortening of the Ti-O bond at this E_g_ vibration mode [38]. Compared with bare TiO_2_ photocatalyst, this weakness of Ti-O bond originated from the oxygen vacancies caused by metallic Cu reduction reaction and Jahn–Teller distortion from incorporation of Cu^2+^ into TiO_2_ crystal [12].

#### 3.1.6. UV–Vis Absorption and Photoluminescence Spectroscopy

**Figure 6:**
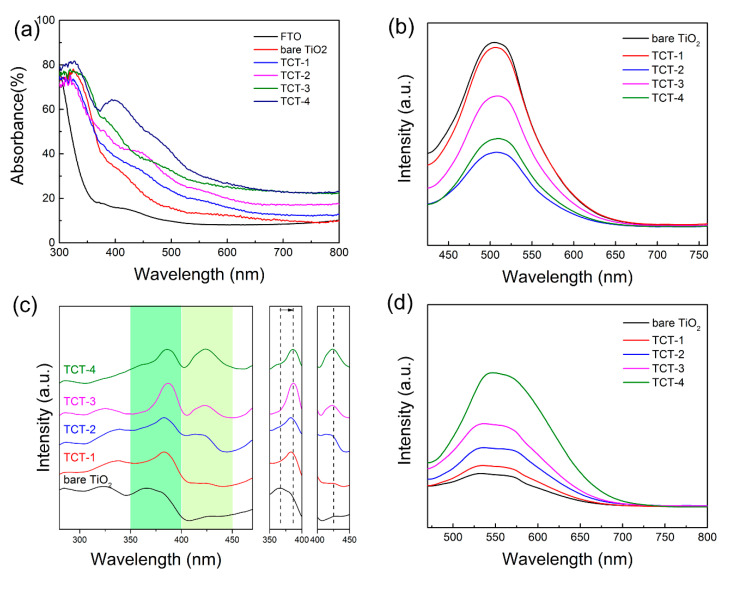
(**a**) UV–vis spectra of FTO substrate, bare TiO_2_ and TCT samples, respectively; (**b**) photoluminescence spectra of all samples with the excitation wavelength at 390 nm; (**c**) PL spectra of all samples with the emission wavelength at 512 nm; and (**d**) PL spectra of all samples with the excitation wavelength at 425 nm.

Figure 6a displays the UV–vis absorption spectra for bare TiO_2_ and as-prepared TCT samples. Almost all samples exhibited main absorbance at UV region. However, compared with pure TiO_2_ materials, TCT samples showed an increasing absorption at visible light region with the thickness of Cu increasing. Without any doubt, the heavy doping of Cu would narrow the band gap of TiO_2_. The thicker Cu film in TCT-4 would even produce Cu_2_O with 2.2 eV band gap [39] from the XRD result. The extending light absorption would improve the photocatalytic property. Furthermore, it should be noted that metal nanoparticles could generate surface plasmon resonance (SPR) effect [40]. Many authors researched embedded noble metal (Au [41,42,43] and Ag [44]) into double TiO_2_ layers to enhance the visible light absorption. In addition, copper nanoparticles could SPR effect at wavelength of around 570 nm. Figure 6a clearly shows that no such absorption peak was present in all TCT samples. This also suggested that the metallic Cu reacted with TiO_2_ sufficiently without metallic Cu leftover.

Photoluminescence spectra were recorded to investigate the migration and recombination of photo-induced carriers. Under excitation at wavelength of 390 nm, the bare TiO_2_ showed strongest emission peak at 512 nm caused by recombination of photogenerated holes and electrons. The heavy doping of Cu led to a weak intensity of emission peak, suggesting much more oxygen vacancies caused by Cu atoms that would serve as trapping sites to capture electrons, which could promote the separation of electrons and holes. Interestingly, the TCT-2 sample had the lowest emission peak intensity, indicating that the moderate amount of doped Cu in TiO_2_ lattice would facilitate efficient separation and migration of charge carriers, thus benefiting photoelectrochemical water oxidation. To classify the effect of doped Cu on the separation of charge carriers, the PL spectra under excitation light with long wavelength was collected. Figure 6c shows the excitation peak with the emission wavelength of 512 nm. The excitation peak at 350–400 nm corresponded to TiO_2_ band gap. The excitation peak of TiO_2_ showed a red shift with the thickness of Cu films increasing, which was ascribed to change of the electron structure by Cu 3d orbitals. These data also prove that the doped Cu narrowed the band gap of TiO_2_ and improved the light absorption. The excitation peak at 425 nm and the emission peak at 550 nm were attributed to Cu_x_O. The intensity of excitation peak rose when the concentration of Cu increased. The presence of this excitation peak disclosed that the thicker Cu film would produce Cu_x_O when reacted with TiO_2_. These results reveal that the balance between doped Cu atoms and oxygen vacancies led to effective separation of electron–hole pairs.

### 3.2. Photoelectrochemical Performance

**Figure 7:**
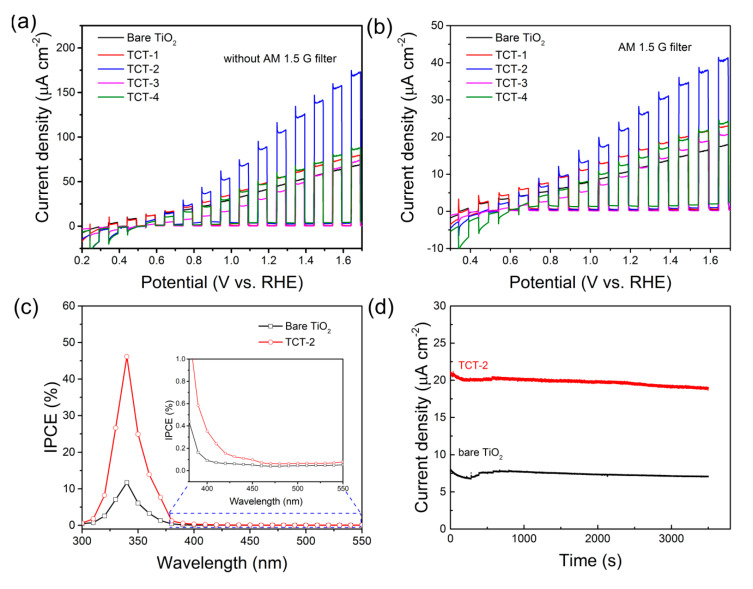
Linear scanning J-V curves of all samples in 0.5 M Na_2_SO_4_ with AM 1.5G filter (**a**) and without filter (**b**); (**c**) IPCE curves measured at 1.23 V (vs. RHE); and (**d**) steady state photocurrent at 1.23 (vs. RHE) for bare TiO_2_ and TCT-2 photoanodes, respectively.

The PEC performance of bare TiO_2_ and as-prepared TCT materials were investigated by measuring the LSV curves in neutral electrolyte under chopped light illumination. As shown in Figure 7a,b, bare TiO_2_ thin film exhibited low photocurrent densities of 11.8 and 45.0 µA cm^−2^ with/without AM 1.5G filter at 1.23 V (vs. reversible hydrogen energy, RHE), while enhanced photo responses were observed for most of TCT anodes, especially the TCT-2 sample. TCT-2 photoanode gave largest photocurrent densities of 28.3 and 108.4 µA cm^–2^ with/without AM 1.5G filter, about 2.39 and 2.41 times higher than bare TiO_2_ thin film, respectively. The huge improvement of photocurrent density might be ascribed to the increased light absorption and enhanced charge separation from the above results. It was found that the doped Cu and oxygen vacancies induced by Cu films had a positive effect on the PEC property for most TCT samples. Moreover, cathodic currents were observed for TCT samples due to the oxygen reduction in the absence of electrons scavengers [45]. Conversely, cathodic current was not observed by bare TiO_2_. These data imply that the doping with Cu (Cu^2+^ and Cu^+^) was beneficial for electrochemical reduction reaction. Besides, all TCT samples displayed an immediate and steady response and recovery towards the on/off cycles of chop light irradiation, revealing the improved PEC performance by introduction of Cu films.

To evaluate the photoconversion efficiency of the TCT materials, incident photon to current efficiency (IPCE) measurements were performed and calculated. Figure 7c shows the IPCE spectra of bare TiO_2_ and TCT-2 sample measured at 1.23 V (vs. RHE). It could be clearly seen that TCT-2 and bare TiO_2_ photocatalysts had the main photocatalytic activity in the UV region. Both samples displayed the highest IPCE at 340 nm with 46.2% and 16.7% for TCT-2 and bare TiO_2_, respectively. This could be mainly attributed to the enhanced separation of photogenerated charge carriers. Furthermore, it should also be noticed that TCT-2 exhibited slight improvement of IPCE near visible light (400–450 nm) compared to bare TiO_2_ photoanode. This improvement might originate from the narrowed band gap of TiO_2_ caused by Cu doping and oxygen vacancies. Above a wavelength of 450 nm, two samples exhibited negligible IPCE values, indicating the poor photocatalytic activity towards long wavelength light. In addition, the steady state photocurrent test for TCT and bare TiO_2_ photoanodes shown in Figure 7d suggested that these two samples showed a stable photocurrent (less than 6% decay) under long time illumination.

**Figure 8:**
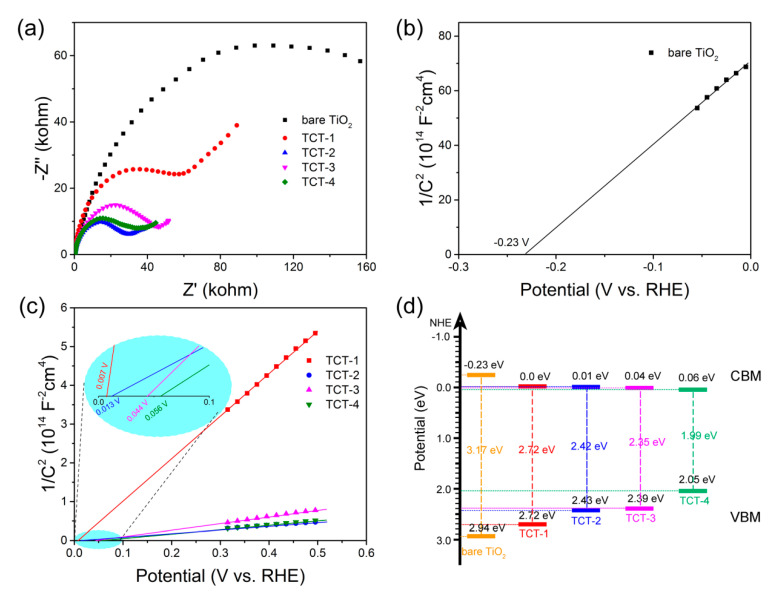
(**a**) EIS curves measured under light illumination; (**b**,**c**) Mott–Schottky plots of bare TiO_2_ and four TCT photoanodes, respectively; and (**d**) band positions of all measured materials.

To further explore the separation and diffusion of photogenerated electrons and holes during the PEC reaction, the EIS and Mott–Schottky curves were collected. As shown in Figure 8a, all TCT samples gave the smaller arc radius than bare TiO_2_, which indicated the fast interfacial charge transport and low charge transport resistance between the electrode and electrolyte. These data demonstrate that the Cu doping could promote the charge separation in photocatalysts by introducing sufficient oxygen vacancies. Meanwhile, TCT-2 sample possessed the smallest arc radius among all TCT and bare TiO_2_ materials, displaying the highest transport efficiency of charge carriers. These results were consistent with the PL behaviors as expected. In MS plots, the E_FB_ could be obtained from Mott–Schottky equation. Figure 7b,c shows that all samples exhibited positive slopes, indicating the n-type feature of all materials. Although the existence of Cu_2_O was revealed from XRD pattern, the MS plot of TCT-4 showed no negative slope as the evidence of p type semiconductor. It might be explained by the small content of Cu_2_O in comparison with TiO_2_ matrix. The charge carrier density was calculated using the MS equation. The obtained values were 1.03 × 10^14^, 2.63 × 10^15^, 3.49 × 10^16^, 1.66 × 10^16^ and 2.64 × 10^16^ cm^–3^ for bare TiO_2_, TCT-1, TCT-2, TCT-3 and TCT-4, respectively. Obviously, the incorporation of Cu atoms into TiO_2_ lattice greatly tuned the concentration of charge carriers in TiO_2_ due to the carriers’ capture and immobilization. The charge carriers’ density of TCT-2 sample was two orders of magnitude higher than that of bare TiO_2_ films, which demonstrated the acceleration of photogenerated carriers’ transfer by doped Cu and oxygen vacancies. The calculated flat band potentials are shown in Figure 8b,c. The E_FB_ of bare TiO_2_ was –0.23 V versus the RHE. The flat band potentials of TCT samples were more positive than that determined for bare TiO_2_, which was due to introduction of Cu 3d orbitals. With the increasing thickness of Cu films, the flat band showed a more positive shift. The band positions are drawn in Figure 8d by the values estimated from VB-XPS (Figure S3) and Mott–Schottky plots, respectively. Indeed, the valence band displayed a negative shift with the increasing of Cu doping, which might be ascribed to the formation of oxygen vacancies induced by Cu reduction and Jahn–Teller distortion. It might be thermodynamically hard for TCT samples to take oxygen evolution reaction compared to bare TiO_2_ counterpart. This was the reason that TCT-3 sample showed a relatively lower photocurrent density compared to bare TiO_2_. The analysis of optical absorption and charge carrier migration demonstrated that all TCT samples were believed to possess better photocatalytic activity than bare TiO_2_ thin films. On the other hand, the narrowing band gap estimated from the valence band maximum (VBM) and conduction band minimum (CBM) extended the photo response to visible light region.

### 3.3. Density Functional Theory Calculations

**Figure 9:**
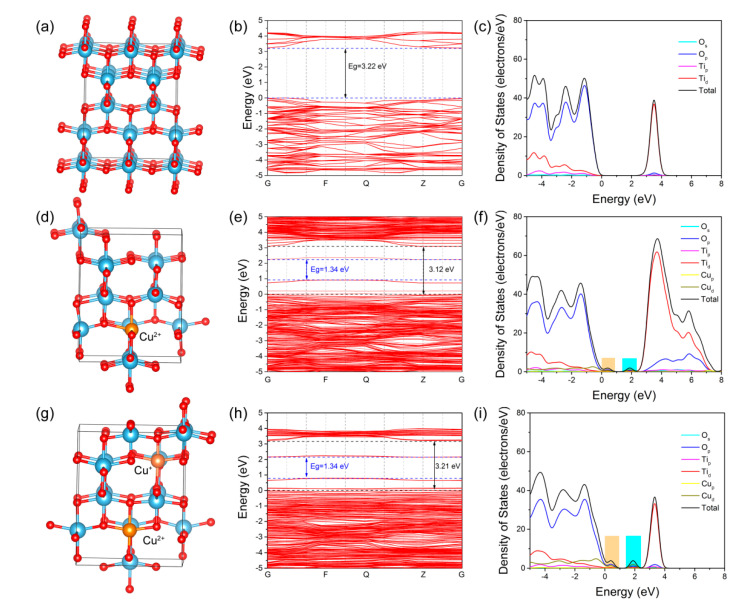
The lattice structure, calculated band structure and density of states (DOS) of: perfect anatase TiO_2_ (**a**–**c**); Cu^2+^-doped TiO_2_ (**d**–**f**); and TiO_2_ with Cu^2+^ and Cu^+^ codoped (**g**–**i**).

To understand the effect of Cu ions and oxygen vacancies on TiO_2_ electronic structure in molecular sight, density functional theory calculations were employed. As shown in Figure 9, anatase bulk TiO_2_ with perfect structure possessed a standard band gap of 3.22 eV. The bare TiO_2_ photoanode in this work showed an energy gap of 3.17 eV, less than the common anatase TiO_2_. This was caused by the introduction of oxygen vacancies in ALD process. The energy gap of TiO_2_ with oxygen vacancies (Figure S4) was calculated to be 2.07 eV from DFT results, due to the existence of defect level near the valence band edge compared to perfect anatase TiO_2_. The formation of oxygen vacancies narrowed band gap of TiO_2_, which enhanced photo absorption in photocatalytic process. Notably, Figure 9f,i clearly shows that the introduced Cu^2+^ greatly modulated the electronic structure of TiO_2_. Two energy levels appeared in TiO_2_ band gap at valence band edge and valence band edge relative to pure TiO_2_. These energy levels were ascribed to the Cu-doping and oxygen vacancies. Oxygen vacancies mainly contributed to the new energy level at valence band edge, while Cu 3d orbitals were mainly responsible for the formation of impurity bands at conduction edge with respect to pure TiO_2._ The resulted Cu-doped TiO_2_ displayed a smaller band gap, giving the possibility of visible light absorption. Furthermore, extra Cu^+^ ions were added into above Cu^2+^-doped TiO_2_ lattice to explore the change of band structures. Interestingly, there were still two kinds of impurity levels and the band gap of materials remained unchangeable, which indicated that the Cu^+^ did not change the position of impurity levels. However, the intensity of energy levels improved by the addition of Cu^+^ compared with the Cu^2+^-doped TiO_2_. In addition, compared to Cu^2+^- and Cu^+^-codoped TiO_2_ and perfect TiO_2_, Cu^2+^-doped TiO_2_ displayed an increasing density of states in conduction band, which would benefit for electrons transport. The DFT results reveal that the moderate doped Cu^2+^ would have a positive effect on TiO_2_ photocatalytic activity by narrowing the band gap and enhancing the charge transport.

**Figure 10:**
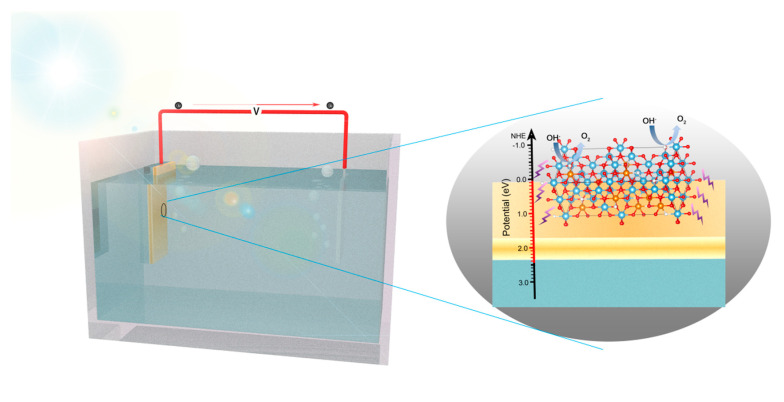
Proposed photocatalytic mechanism of TCT-2 sample under light irradiation.

Combining the experimental and theoretical results, the proposed photocatalytic mechanism of TCT-2 samples is illustrated in Figure 10. Through copper reduction, doped Cu and abundant oxygen vacancies in TiO_2_ brought in impurity and defect levels, which shifted the CBM and VBM to 0.01 and 2.43 eV, respectively. These extra energy levels obviously decreased the band gap of TiO_2_ (2.42 eV) and promoted light absorption region to visible light. Besides, the formation of oxygen vacancies could trap electrons and improve the separation and transfer of charge carriers. In the case of oxygen vacancies on the surface, they were favorable for OH^–^ adsorption and could serve as active sites for oxygen evolution reaction. Based on the above analysis, the doping Cu ions and oxygen vacancies induced by Cu incorporation could significantly enhance the photo response and facilitate the photo-generated charge carriers’ separation and transfer, evidenced by experimental results as well as DFT calculation.

## 4. Conclusions

In this study, we prepared TiO_2_/Cu/TiO_2_ sandwich structure. Through annealing under Ar atmosphere, the Cu-doped TiO_2_ with abundant oxygen vacancies formed. X-ray photoelectron and Auger spectra revealed the existence of doped Cu and oxygen vacancies. With the thickness of core Cu layer increasing, the number of oxygen vacancies and Cu^+^ grew. The core copper could promote the formation of Cu^2+^, Cu^+^ and oxygen vacancies by metal reduction and Jahn–Teller distortion. The resulted V_o_-rich Cu-doped TiO_2_ exhibited an enhanced light absorption due to the introduction of impurity levels in TiO_2_ band gap, which was demonstrated by DFT calculations and UV–vis spectra. Furthermore, the PL spectra and EIS analysis indicated that the efficiency of charge carriers’ separation and transfer in-doped TiO_2_ was improved because of the electron capture by oxygen vacancies. In addition, the exceeding concentration of Cu^+^ would consume the electrons and impede the improvement of photocatalytic property. As a result, the moderate Cu-doped TiO_2_ exhibited an enhanced photoelectrochemical performance, about 2.4 times higher than undoped TiO_2_ materials. This work provides an efficient strategy to prepare metal- and non-metal-doped semiconductors with the simultaneous introduction of vacancies through heat reduction reaction, which leads to an improved photocatalytic performance.

## Figures and Tables

**Table 1:** The thicknesses of as-prepared bare TiO_2_ and four TCT thin films.

Samples	Bare TiO_2_	TCT-1	TCT-2	TCT-3	TCT-4
Thickness (nm)	104.74 ± 1.63	115.18 ± 2.67	124.13 ± 1.40	135.26 ± 1.42	144.59 ± 1.27

## Supplementary Materials

The following are available online at https://www.mdpi.com/1996-1944/13/19/4326/s1, Figure S1: the atomic dispersion of TCT-4 from the line scanning model, Figure S2: XPS survey spectra of bare TiO_2_ and all TCT samples, respectively, Figure S3 XPS Valence band spectra of bare TiO_2_ and all TCT samples, respectively, Figure S4: The lattice structure, calculated band structure, and density of states (DOS) of TiO_2_-V_o_, Figure S5: The transmittance (a) and reflectance (b) of five samples from UV–vis measurement.
