# Supplementary material for: Enhancement of Titania Photoanode Performance by Sandwiching Copper between Two Titania Layers
Source: Materials (Basel). 2020 Sep 28;13(19):4326. doi: 10.3390/ma13194326 (PMC7579609; doi:10.3390/ma13194326)
Supplement: Supplementary file 1 [file materials-13-04326-s001.pdf]

materials

Supplementary

Enhancement of Titania Photoanode Performance by Sandwiching Copper between Two Titania Layers

Fan Yang [1,2], Ruizhuang Yang [1], Lin Yan [1], Jiankun Wu [1], Xiaolin Liu [1,2], Lirong Yang [1], Minglong Zhong [1], Xuan Luo [1,*] and Lin Zhang [1,2]

1 Research Center of Laser Fusion, China Academy of Engineering Physics, Mianyang 621900, China; yangf711@mail.ustc.edu.cn (F.Y.); rzyang99@mail.ustc.edu.cn (R.Y.); yanlin77@mail.ustc.edu.cn (L.Y.); wujiankun19@163.com (J.W.); lxl91003@mail.ustc.edu.cn (X.L.); yanglirong83@163.com (L.Y.); 13110200008@fudan.edu.cn (M.Z.); zhanglin@caep.cn (L.Z.)

2 Department of Physics, University of Science and Technology of China, Hefei 230026, China

* Correspondence: luoxuan@caep.cn

Received: 6 August 2020; Accepted: 10 September 2020; Published: date

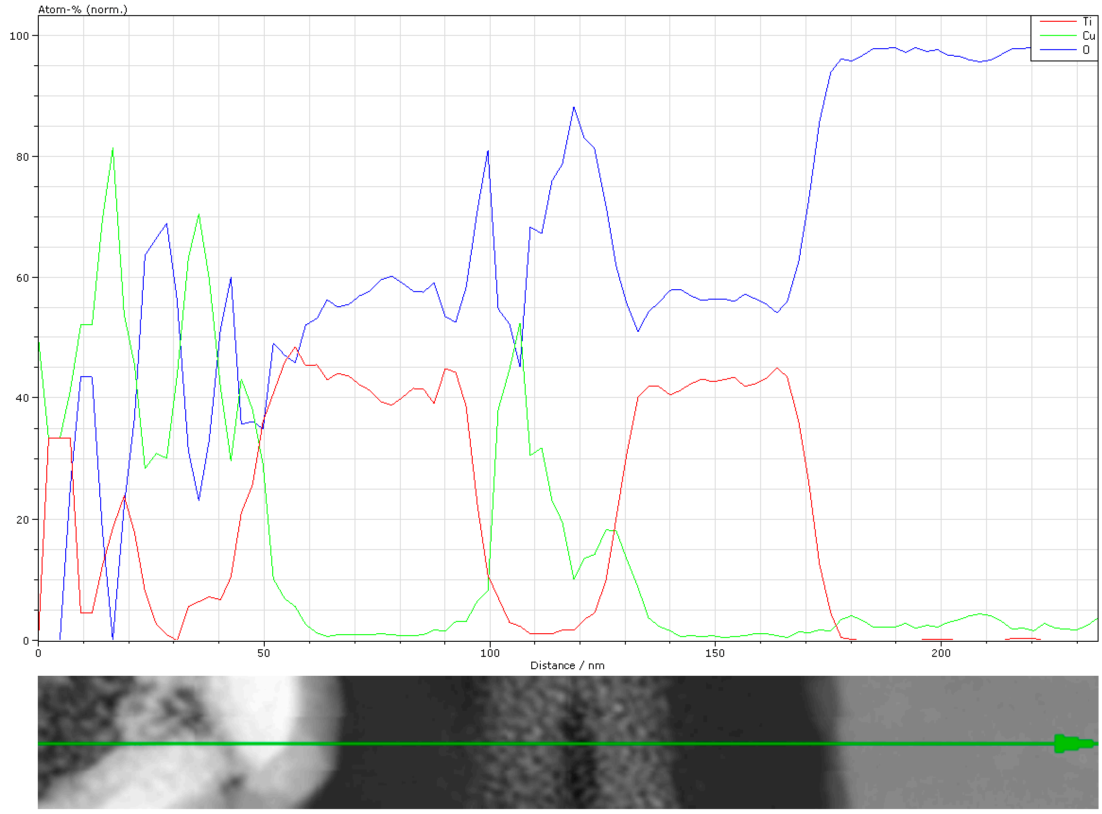

Figure S1. the atomic dispersion of TCT-4 from the line scanning model.

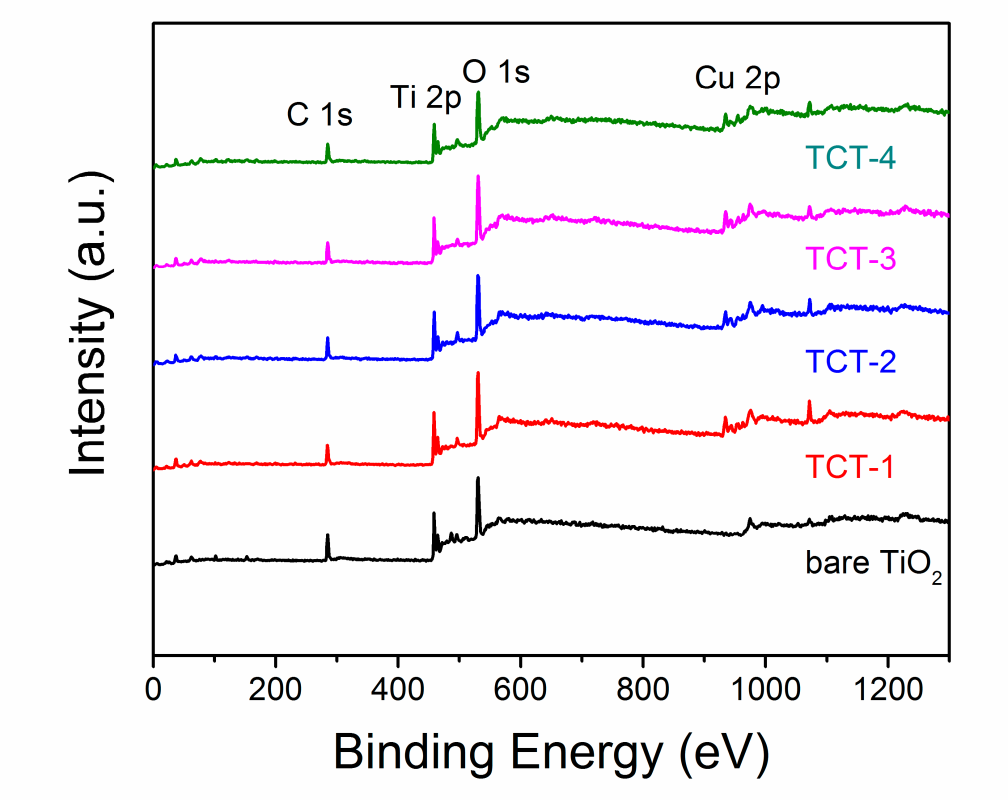

Figure S2. XPS survey spectra of bare TiO_2 and all TCT samples respectively.

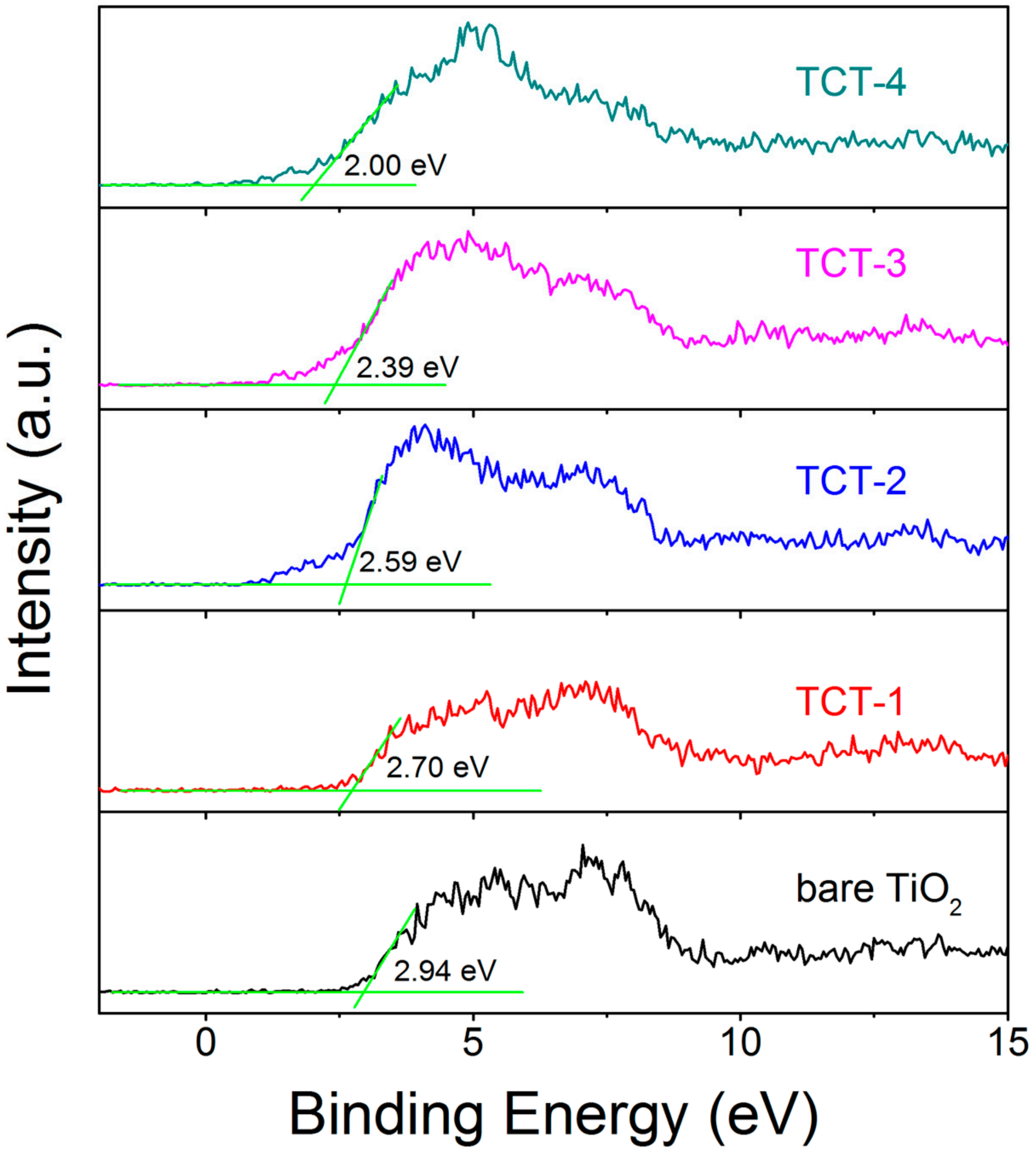

Figure S3. XPS Valence band spectra of bare TiO_2 and all TCT samples respectively.

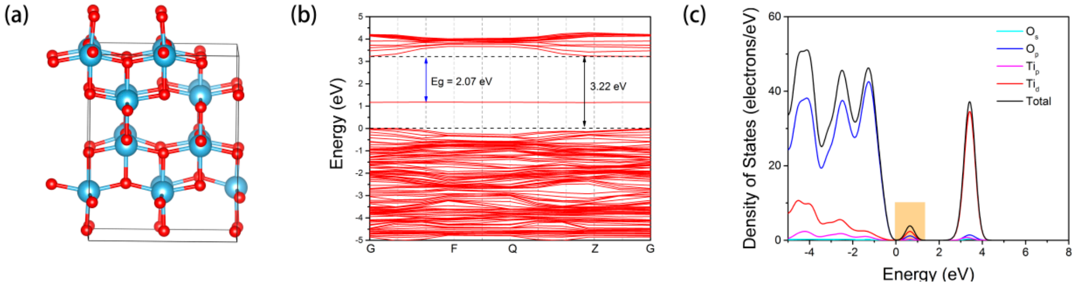

Figure S4. The lattice structure, calculated band structure and density of states (DOS) of TiO_2-Vo.

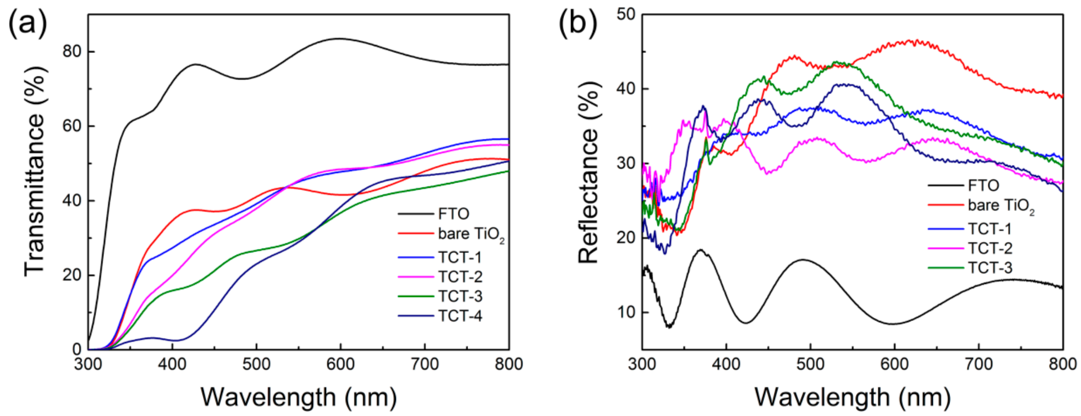

Figure S5. The transmittance (**a**) and reflectance; (**b**) of five samples from UV-vis measurement.

The absorptance spectra was calculated by the Equation:

$$A = 1\text{-}T\text{-}R \quad (1)$$

Calculations

The IPCE can be calculated by the Equation:

$$\text{IPCE}(\%) = (1240 \times J_{ph})/(\lambda \times P_{in}) \quad (2)$$

where Jph is the measured photocurrent density of the experimental sample, λ is the wavelength and Pin is the intensity of the incident light.

Mott-schottky Equation:

$$\frac{1}{C^2} = \frac{2}{N_D e \varepsilon_0 \varepsilon}(E - E_{FB} - \frac{kT}{e}) \quad (3)$$

Where C is the capacitance, N_D is the charge carrier density, e is the elemental charge, ε is the relative permittivity of material, ε_0 is vacuum permittivity, E is the applied potential in electrochemical measurement, E_{FB} is the flat band potential, k is the Boltzman constant and T is measurement temperature.

© 2020 by the authors. Submitted for possible open access publication under the terms and conditions of the Creative Commons Attribution (CC BY) license (http://creativecommons.org/licenses/by/4.0/).
